# Supplementary material for: A Debittered Complex of Glucose-Phenylalanine Amadori Rearrangement Products with β-Cyclodextrin: Structure, Molecular Docking and Thermal Degradation Kinetic Study
Source: Foods. 2022 Apr 29;11(9):1309. doi: 10.3390/foods11091309 (PMC9105382; doi:10.3390/foods11091309)
Supplement: Supplementary file 1 [file foods-11-01309-s001.zip › foods-1689826-supplementary.pdf]

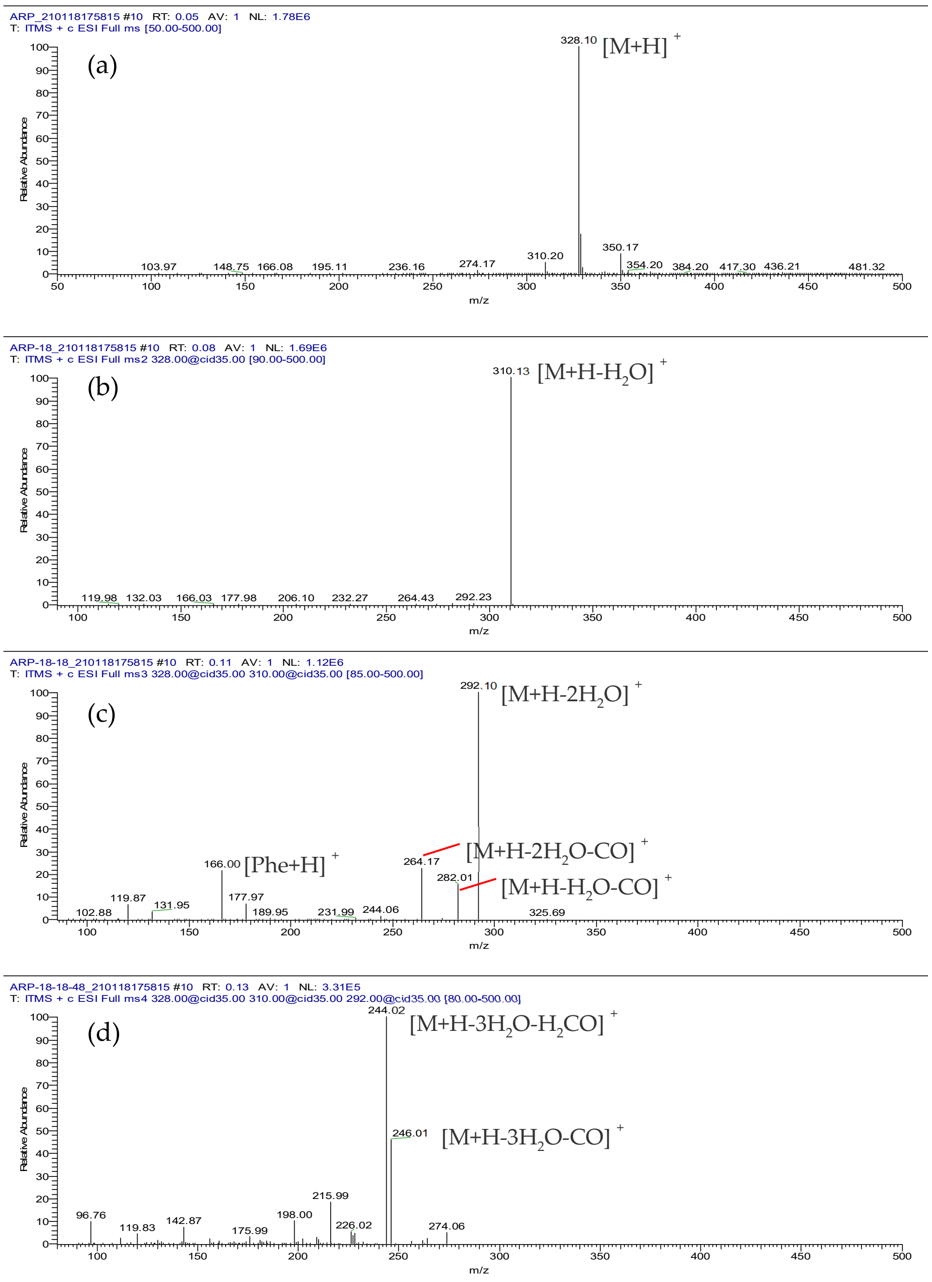


**Figure S1.** (a) MS, (b) MS2, (c) MS3 and (d) MS4 spectra of purified Glu-Phe ARPs**.**

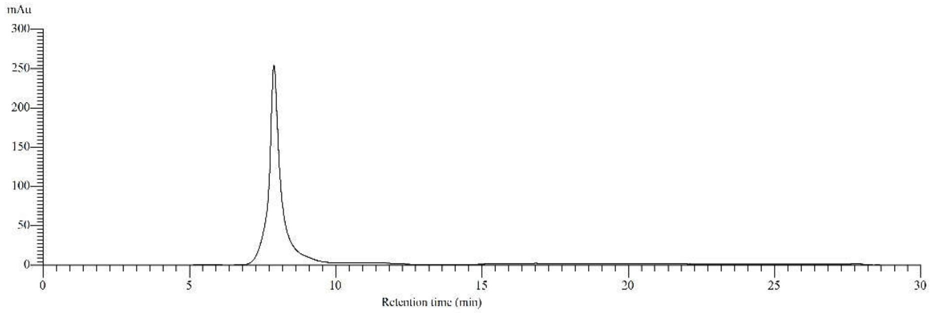


**Figure S2.** HPLC-DAD chromatogram of purified Glu-Phe ARPs.

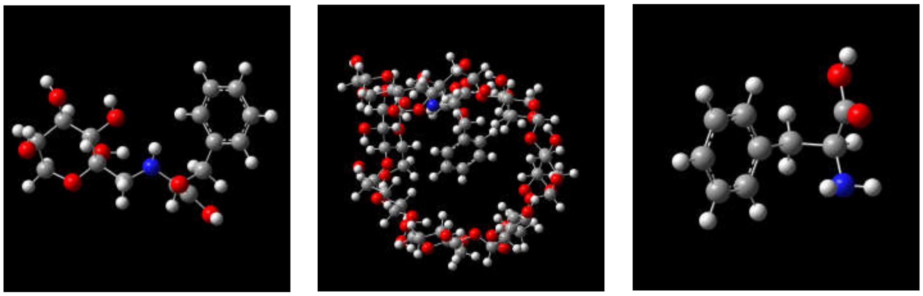

(a) (b) (c)

**Figure S3.** The optimized structures of (a) Glu-Phe ARPs, (b) CD-ARP complex and (c) Phe.
